# Investigating movement synchrony in therapeutic settings using socially interactive agents: an experimental toolkit

**DOI:** 10.3389/fpsyt.2024.1330158

**Published:** 2024-02-05

**Authors:** Janet Wessler, Patrick Gebhard, Sigal Zilcha-Mano

**Affiliations:** ^1^ Affective Computing Group, Cognitive Assistants, German Research Center for Artificial Intelligence, Saarbruecken, Germany; ^2^ Psychotherapy Research Lab, Department of Psychology, University of Haifa, Haifa, Israel

**Keywords:** synchrony, psychotherapy, working alliance, socially interactive agents, machine learning, experimental design

## 1 Introduction

### 1.1 Parameters and measurement of interactional synchrony

Movement synchrony is the temporal coordination of postures and gestures between interaction partners (1). In contrast to mimicry — which is defined as an unconscious imitation of specific gestures and expressions (e.g., both interaction partners scratch their head) — synchronous movements can take the same or different form as long as they are time-coupled (e.g., one partner scratches the head and the other the leg).

Five general criteria can be used to describe interactional synchrony (2). First, synchrony takes place in a certain *context*, which could for example be affiliative or competitive. Second, synchrony occurs within a certain *modality*, e.g. movements or verbal expressions. Third, within a modality, interactants use specific *resources* for synchrony, e.g., postures or gestures within the movement modality. Fourth, synchrony is characterized by *entrainment*, that is, who is leading or following the synchrony. Fifth, synchrony occurs with different *time lags*, from 0 seconds and perfect matching up to a few seconds. However, the exact parameters of time lag for synchrony vary between studies, for example 5 seconds (3) or 2.5 seconds (4). Thus, currently, it remains an open question which time lag of synchrony is the most appropriate for certain contexts and modalities (2, 5). Moreover, Scheidt and colleagues (2) specified eight dimensions which are specific to movement synchrony movement, that is, its spatial directions (e.g., towards or far from the other), its amplitude, sinuosity, duration, event structure, phase shifts, frequency in a given time period, and the content of synchrony (e.g., same versus different body parts).

Investigating such temporal dynamics of nonverbal behaviors between human interaction partners is complex and typically involves immense human resources and time efforts. Participants are typically video-recorded, and the interactions are annotated by blind coders. In recent years, developments in technology partially allowed for automatic analysis of certain behaviors using software like OpenFace (6), OpenPose (7) or Movement Energy Analysis (MEA; 8–11). Through certain algorithms, such software quantifies the amount of nonverbal behavior which is present in the videos, thus facilitating the measurement of synchrony profoundly. In psychotherapeutic research, movement synchrony is typically measured by MEA (3, 8). Computer algorithms calculate the movement energy as the amount of pixel changes between picture frames. Then, cross-correlations between this quantified movement energy are calculated to find covariations in the movements between interactants within a time-lag of between 0 sec and 10 sec. Then, these synchrony values are compared against levels of so-called pseudo-synchrony, in which random subsets of the interaction are paired and cross-correlations are computed.

### 1.2 Effects of synchrony on the dyadic relationship

In the past few years, a great interest emerged in measuring movement synchrony between the patient and therapist in psychotherapy because it is assumed to represent an important component of the therapeutic relationship, or even serve as its underlying mechanism (12). In the Interpersonal synchrony (In-sync) model of psychotherapy (12) movement synchrony between interaction partners and inter-brain coupling between them mutually influence each other positively. The process of alignment takes place within a time frame of a few milliseconds to 10s via a simple association between perception and motor movements. The inter-brain coupling then facilitates higher cognitive processes that involve language and reasoning. These processes lead to alliance formation and strengthening — a positive working relationship between patient and therapist.

So far research had assumed that the synchrony-outcome relationship is linear, that is, more synchrony leads to better outcomes, even though this relationship is small (13, 14). However, recent arguments claim that this might not be always the case (1). However, recent arguments claim that this might not be always the case (15). More synchrony could also lead to adverse, unwanted effects. This is due to the fact that humans are not only driven by a need to affiliate and belong (e.g. 16–18), but similarly by a need for uniqueness, autonomy, and distinguishness (e.g., 19). Thus, when patients are rather in search for independence from others, the feeling of being in sync and aligned with the therapist might have adverse effects on the therapeutic outcome (1).

Several studies showed a positive association between movement synchrony and working alliance and positive treatment outcomes during psychotherapy (3, 8, 20–22). However, whereas in some studies the effect reached significance (e.g., 3, 23), in others it did not (e.g., 7, 24). Yet other studies reported that no association exists, or even that higher synchrony is associated with a poorer process and outcome of treatment (25–27).

Focusing on the association between the strength of the patient-therapist working alliance and the level of synchrony between them, a similar pattern of mixed results emerges. Many of the studies examining the association between synchrony and alliance found a positive association, so that higher synchrony was associated with stronger alliance; in some, the effect reached significance (e.g., 20, 23, 28), whereas in others, it did not (e.g., 29). Furthermore, three studies found a negative association (25, 30, 31), with a stronger alliance being associated with lower levels of synchrony.

In summary, the existing line of research remains rather inconclusive about which parameters of movement synchrony exactly drive its positive effects, if it has always positive effects, and which factors might drive negative effects of movement synchrony — more conclusive studies are needed (2).

One of the main drawbacks of the current studies is that they are mainly correlational in nature. The manipulation of synchrony in psychotherapeutic settings usually is not feasible, either from an ethical point of view or due to the study designs. Moreover, it would have to involve excessive training of confederates to be able to react with standardized levels of synchrony towards every participant.

However, in order to investigate causal relationships instead of (cross-)correlations between nonverbal behaviors and higher-level outcomes of the interaction, it is necessary to manipulate certain parameters of nonverbal behaviors. For example, mimicry research has relied on human confederates to manipulate the amount of mimicry between interactants (32). Although human confederates should be extensively trained before conducting such studies, a certain variance between interactions can occur, confederates might not be blind, and an experimental manipulation of fine-grained parameters like the time-lag of the imitation is difficult (see 33). Due to these challenges, mimicry research has employed socially interactive agents (SIAs; 34) in order to keep the mimicry manipulation as controlled as possible (33, 35). In general, using virtual reality has many advantages for psychological experiments (36). SIAs have a physical representation, which is similar to but does not equal humans. They can detect social signals like smiles and movements, analyze them and react with similar behaviors themselves with a certain time-lag, that is, engage in mimicry. The reasoning behind such research is that some equivalence between humans and computers is assumed in human-computer interaction (37), manipulating nonverbal behaviors of SIAs in human-SIA interaction could serve as a proxy for studying consequences of nonverbal behaviors in human-human interactions. Thus, it was for example possible to experimentally test the effects of time-lags of 1 sec vs. 3 sec mimicry from either ingroup are outgroup SIAs on interpersonal outcomes like liking and trust (33). Still, other research on interpersonal coordination like movement synchrony has not yet explored the possibility of using SIAs as an experimental tool. There is a need for a virtual testing environment to experimentally test synchrony-related hypotheses (36). With the proposed conceptual model, we aim at creating an experimental framework using SIAs which then can be used to fill these research gaps.

## 2 Building an experimental toolkit with SIAs

**Figure 1:**
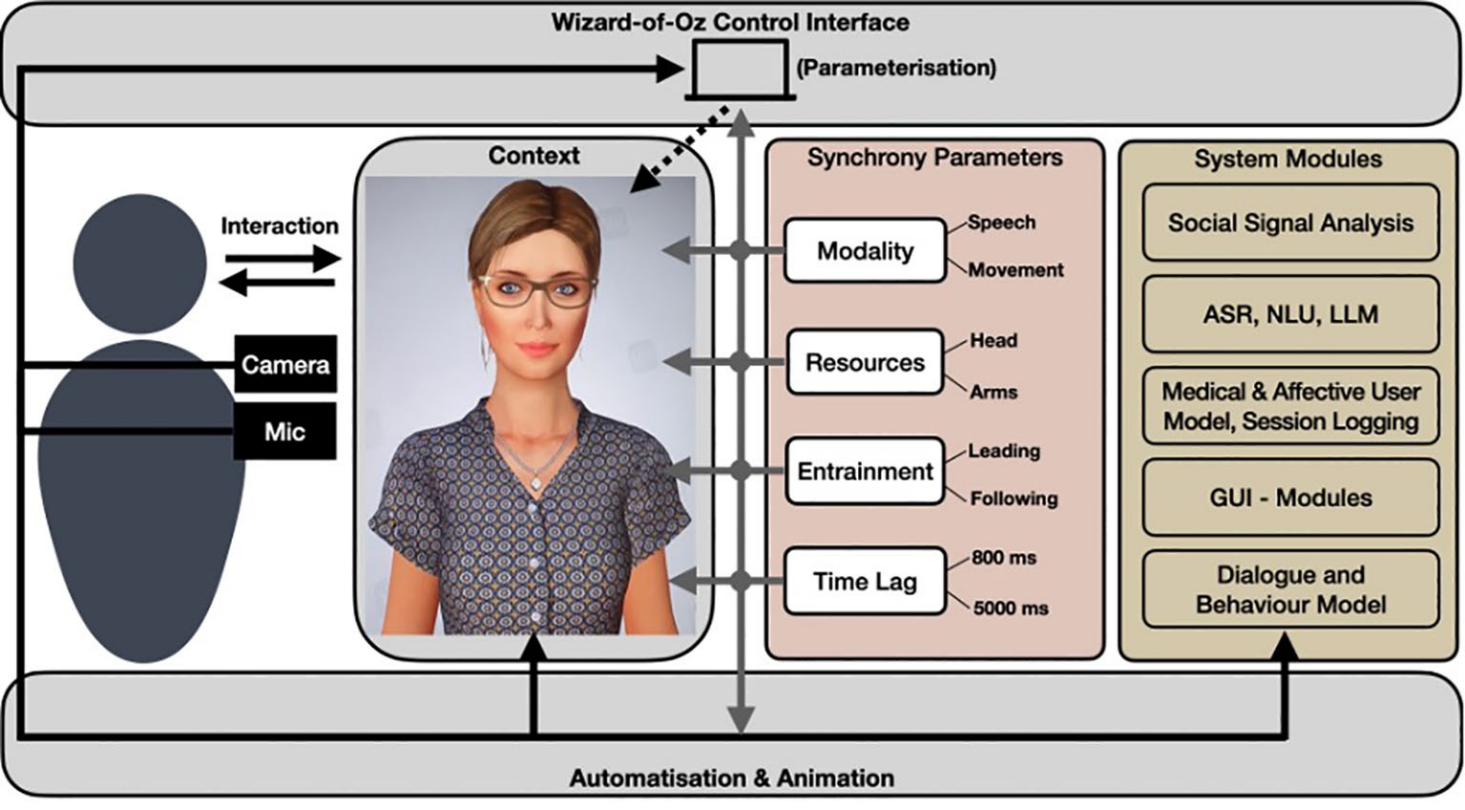
Overview of the architecture and modules of the experimental toolkit for studying synchrony between humans and socially interactive agents. SIA obtained from Charamel, https://www.charamel.com.

As an experimental toolkit for exploring synchrony and related concepts, we propose a highly parameterisable real-time architecture using a SIA. To model the SIA’s synchrony behavior, we will use both theory-driven and data-driven approaches. For example, data collected from thousands of therapeutic sessions can be used for machine learning (38, 39). This real-time architecture then allows to configure and support relevant aspects such as context (e.g., competitive or affiliative), modality (speech, movement), resources (head, arm), entrainment (leading, following), and time lag (delay in milliseconds). **Figure 1** shows an overview of the proposed experimental toolkit.

The toolkit allows to study synchrony with an SIA in a fully automated mode up to fully human controlled (wizard). In each mode, synchrony parameters (**Figure 1**, middle) can be manipulated at any time. The automated mode relies on the following additional technological SIA components (34) (**Figure 1**, right side), such as social signal analysis, automatic speech recognition (ARS), natural language understanding (NLU), large language models (LLM) (40), GUI modules for questionnaires, and user models (affect, medical) which drive together the dialogue and behavior models for a (configurable) real-time behavior rendering of the SIA. LLMs are used to create context information which is integrated into existing authoring frameworks (e.g., 41) for SIAs for further adaptation and verifications by experts. In each mode, relevant timing information, such as user (reactions), module processing time, and the participants’ signals (voice, video) are recorded for further data analysis.

When investigating movement synchrony with SIAs, time delay, and intensity are essential aspects that are carefully controlled by the proposed experimental toolkit. Controlling the time delay involves thoroughly understanding important parameters and the data and information processing pipeline. In the autonomous mode, a significant movement (e.g., head nodding) has to be detected by social signal classifiers before any observable animation as a reaction of the SIA is shown. Usually, there is a defined configurable movement threshold. The movement range (e.g., head tilt angle from 2 – 10 degrees) correlates to the intensity (e.g., weak - strong head nodding). Usually, the detection takes between 50 – 200 ms depending on which modalities (face, head, body, or any combination of them) are involved and which movement thresholds are defined. After that period, a suitable action is selected, and after a defined delay is shown by the SIA. The delay is usually between 500 – 2000 ms (or longer), and the observable reaction of the SIA usually takes as long as the detected social signal but can be altered concerning timing and intensity. The minimal system intrinsic reaction time is between 100 – 400 ms + 20 ms (max. approximated) inter software module delay. In general, an important toolkit feature is accurately logging such events for future analysis. This is especially important if a human wizard does the detection of social signals and the selection of the movement modality.

## 3 Exemplary clinical study

Let us imagine depressive patients use the toolkit in a daily diary study in which they interact with the SIA for about 10 minutes over 8 days via an App on a mobile device at home (42). The SIA asks the patient about his/her day and how s/he feels. The dialogue model represents the questions based on a specific therapy model. While listening, the toolkit analyses the patient’s social signals (e.g., smiles, head movements) and recognizes the speech content via ASR and NLU. At best, the ASR and NLU are trained for the specific requirements to improve their accuracy. LLMs (retrained for the specific situation) are used for an automatic contextual annotation that might be useful at later stages for an automatic generation of synchrony behavior. On random days, patients are exposed to the *no-delay* condition: the SIA synchronizes with the patient’s movements within typical technological limits (between 200-1000 milliseconds). On the other days, patients are randomly exposed to the *3 sec-delay, 5 sec-delay*, or *7 sec-delay* synchrony conditions: the SIA synchronizes with the patient’s movements after a delay of 3, 5, or 7 sec. Synchronizing here means that the SIA might not mimic the exact movement of the patient, but, for example, might move their arm when the patient moves the head (i.e., modality switch). The agent’s behavior is driven by rule-based, learned, or hybrid computational behavior models or controlled by a wizard (WOZ control interface). Afterward, the patient will indicate their alliance with the SIA during the interaction in a questionnaire, including their general rapport and how much they trusted them.

Moreover, before and after the eight days of interaction a patient’s symptoms will be assessed to measure the interaction outcome. Thus, we can test the hypothesis if there is an optimal level of synchrony for its positive effects to occur in this setting (5). Moreover, we can explore whether the optimal level is individual-specific or more universal. If the optimal level is universal, it will be possible to train therapists (real and virtual) to synchronize with their patients at this optimal level. If the optimal level is individual-specific, it will be possible to provide individuals with feedback to raise awareness of their optimal level. It will also be possible to provide therapists with training on how to identify the optimal level of each of their patients and how to optimize the synchrony level accordingly.

## 4 Possible future research questions

With the proposed experimental toolkit, psychotherapeutic researchers have the possibility to experimentally investigate various types of currently open research questions. For example, one important question to investigate is how specific timing lags of synchrony influence the working alliance between a SIA as a therapist and the user. One possibility here is that the relationship between time lag and working alliance is invertedly U-shaped (Wessler et al., under review): very low (e.g., 1 sec) and very high (e.g., 8 sec) time lags lead to lower levels of working alliance compared to moderate time lags (e.g., 3-5 sec). Also, such a curvilinear relationship might be shifted by interindividual preferences and interpersonal tendencies. One such interpersonal tendency in the context of synchrony could be a person’s attachment style (16, 18). While anxiously-attached individuals might prefer rather short time delays, avoidant individuals might prefer longer time delays for a positive working alliance with their therapist. Even more, such preferences might be sensible to change throughout the course of psychotherapy. Results of possible future studies testing such questions would help to clarify the theorizing about interactional synchrony in therapeutic settings and to derive practical recommendations for psychotherapists.

## 5 Conclusion

Here we outline the necessity to build an experimental toolkit to further investigate the open research questions about the relationship between movement synchrony and therapy-related outcomes. We propose an experimental toolkit in which certain parameters of synchrony behaviors of SIAs can be specifically manipulated in a controlled manner, such as time-lag. Such a toolkit would facilitate the resource-intensive character of synchrony research, enable experimental manipulations, and help to derive practical recommendations.

## Author contributions

JW: Conceptualization, Project administration, Writing – original draft. PG: Conceptualization, Software, Supervision, Writing – review & editing. SS: Conceptualization, Supervision, Writing – review & editing.
